# Perioperative predictors of prolonged length of hospital stay following total knee arthroplasty: a retrospective study from a single center in China

**DOI:** 10.1186/s12891-020-3042-x

**Published:** 2020-01-31

**Authors:** Xiaoxiao Song, Caiwei Xia, Qiangqiang Li, Chen Yao, Yao Yao, Dongyang Chen, Qing Jiang

**Affiliations:** 1 Department of Sports Medicine and Adult Reconstructive Surgery, Nanjing Drum Tower Hospital, School of Medicine, Nanjing University, 321 Zhongshan Road, Nanjing, 210008 Jiangsu People’s Republic of China; 2 Department of Orthopedics, Taikang Xianlin Drum Tower Hospital, Nanjing University School of Medicine, Nanjing, Jiangsu People’s Republic of China; 3 Department of Sports Medicine and Adult Reconstructive Surgery, Nanjing Drum Tower Hospital, Clinical College of Nanjing Medical University, Nanjing, China; 4 Laboratory for Bone and Joint Diseases, Model Animal Research Center, Nanjing University, Nanjing, 210093 Jiangsu People’s Republic of China

**Keywords:** Length of hospital stay, Total knee arthroplasty, Operative time, Predictor

## Abstract

**Background:**

Decreasing the length of hospital stay is an ideal course of action to appropriately allocate medical resources. The aim of this retrospective study was to identify perioperative factors that may decrease the length of hospital stay (LOS).

**Methods:**

In this study, we collected the data on 1112 patients who underwent primary total knee arthroplasty surgery (TKAs) at our institution from Jan 1, 2011 to Nov 31, 2017. Based on the published literature, 16 potential factors (12 preoperative variables, 1 intraoperative variable, and 3 postoperative variables) were investigated. The patients requiring a hospital stay longer than the mean LOS (8 days) were defined as patients with a prolonged LOS. The factors with a *P* value less than 0.1 in the univariate analysis were further analysed in a multivariate model. An ordinal regression was used to determine independent risk factors for a prolonged LOS.

**Results:**

The mean LOS was 8.3 days (±4.3), with a range of 2 to 30 days. Sixteen variables were analysed by univariate analysis, and 11 of them had *p* < 0.1 and were included in the multivariable model. Finally, 9 factors were found to be associated with a prolonged LOS. Among the 9 variables, 2 were surgery-related factors (operative time and intraoperative blood loss), and 3 were patient-related factors (age, ASA classification and neurological comorbidities).

**Conclusion:**

In this study, we found that the clinical protocol, complications, the patient’s age, the ASA classification, neurological comorbidities, the operative time, the ward, intraoperative blood loss and the surgeon were all factors contributing to a prolonged LOS. In clinical practice, these factors provide important information for the surgeon and are useful for identifying patients with a high risk of a prolonged LOS.

## Introduction

Total knee arthroplasty (TKA) is a highly effective procedure for relieving pain and improving knee function for people suffering from advanced knee rheumatoid arthritis (RA) and osteoarthritis (OA) [1]. With the rise in healthcare costs, the demand for TKA surgery recently increased due to factors such as the ageing population, improvement in the standard of living, pursuit of a high quality life, and the maturity of TKA surgery [2]. In addition, patients sometimes have to wait to be treated for days or even months and are not treated in a timely manner due to limited hospital resources [3]. To appropriate allocate resources, decreasing the length of hospital stay (LOS) is an ideal course of action [4].

In recent decades, there has been a considerable amount of progress in reducing the LOS [5] because many potential factors for prolonged LOS have been studied, including age, sex, comorbidities, weekend admission, the American Society of Anesthesiologists (ASA) classification, preoperative education, and the need for blood transfusion [6–13]. Additionally, with the introduction of fast-track protocols, which emphasize pain and blood management, limit perioperative complications, and promote postoperative early ambulation, the LOS has decreased dramatically without compromising functional recovery [4, 6].

However, fast-track protocols are not easy to implement in China for the following two reasons. First, as China is a developing country, extended care facilities such as skilled nursing facilities and rehabilitation units are not as prevalent in China as they are in developed countries [14]. Additionally, the roles of doctors and nurses in China are demanding; they do not have a sufficient amount of time to address discharged patients. Second, the relationship between doctors and patients is sometimes tense [15, 16], and patients often request a prolonged hospital stay to ensure that they can perform daily activities. This phenomenon is also common in other developing countries [7]; however, few studies have predicted the factors that influence LOS in this medical environment. Thus, we performed this study to determine which factors can significantly affect LOS after TKAs in this medical environment.

## Materials and methods

All patients undergoing primary unilateral TKA in our hospital between Jan 1, 2011 and Dec 31, 2017 were enrolled in this study. Based on the previous literature, the following 16 potential factors were recorded: age, sex, clinical protocol, operative time, surgeon, postoperative complications, day of surgery, payment type, Barthel Index (BI) score, preoperative haemoglobin (Hb), intraoperative blood loss, range of motion (ROM), transfusion, ASA classification, comorbidities and ward. All data were extracted by one author.

All TKAs were performed by four experienced orthopaedic surgeons. General anaesthesia and a tourniquet inflated to 300 mmHg were used for all patients before the surgery started. As a routine practice, 100 ml of cefazolin was administered intravenously approximately 30 min before the surgery. All TKAs were performed by the medial parapatellar approach. One drain was placed in the articular cavity after the surgery and was removed one to 2 days later. Postoperatively, a multimodal pain control medication, including 50 mg of tramadol and a 50 mg injection of flurbiprofen axetil or 40 mg parecoxib sodium, was administered to all patients two times a day. Beginning in 2014, a new clinical protocol, which mainly included the following 3 processes, was performed: 1) building a special rehabilitation team aimed at helping patients regain function until they achieved the discharge criteria; 2) introducing a “cocktail pain control” model, including the injection of 10 mg morphine and 150 mg ropivacaine around the incision after the wound was closed; and 3) implementing sound perioperative management plan to prevent complications, which included the administration of 50 mg of tranexamic acid intravenously to all patients after implantation to reduce blood loss and the routine administration of chemoprophylaxis (rivaroxaban or low molecular weight heparin) and mechanical prophylaxis (pneumatic compression with a foot pump) to all patients to prevent deep vein thrombosis (DVT).

Patients were permitted to be discharged after they met the following criteria: (1) safe and independent walking with a walking aid; (2) flexion angle of the knee ≥90°; (3) no complications or controlled complications; (4) adequate pain control with a VAS score of < 3 at rest and < 5 during mobilization; and (5) strongly demands to be discharged. Our therapists evaluated whether a patient had met the discharge criteria, discussed this evaluation with the surgeon, and finally, both of them determined whether the patient should be discharged.

All LOS values mentioned in this article correspond to the duration from the day of surgery to the day of discharge [13, 17]. The patients requiring a hospital stay longer than the mean LOS (8 days) were regarded as patients with a prolonged LOS. In this study, we collected data from the medical database, and patients’ private information was not retrieved. Therefore, consent for participation was waived.

### Statistical analysis

Statistical analyses were performed with SPSS version 23.0 (SPSS Inc., Chicago, IL, USA). Firstly, we performed exploitative analysis to detect the collinearity and avoid obvious internal correlation among the 16 variables. Then, in the univariate analysis, we used one-way ANOVA to describe the associations between continuous variables and LOS and the chi-squared test to describe the associations between categorical variables and LOS. The risk factors with a *p* value less than 0.1 in the univariate analysis were used in the multivariate analysis. In the multivariate analysis, we used binary regression to determine the independent risk factors for increased LOS. Odds ratios and 95% confidence intervals (CIs) were reported. A p value less than 0.05 was used to determine statistical significance.

## Results

In the exploitative analysis, no collinearity was found. The demographic data are presented in Table 1. A total of 1112 patients were included in this study. There were 927 (83.5%) females and 183 (16.5%) males. The mean age was 67.9 years, and the age range was from 28 to 89 years. The mean LOS for all patients was 8.3 ± 4.3 days and ranged from 2 to 30 days.

**Table 1:** Demographic features of 1112 patients who underwent total knee arthroplasty surgery

Variables	Sample, N (%)	Mean ± SD	Mean LOS (days)	*P*-value
Age (years)		67.9 ± 7.9		0.036
≤ 65	396 (35.7)		7.9 ± 2.4	
>65	714 (64.3)		9.5 ± 3.6	
Gender				0.441
Female	927 (83.5)		8.3 ± 4.3	
Male	183 (16.5)		8.0 ± 4.2	
Clinical protocol				< 0.01
Traditional protocol	71 (6.4)		11.6 ± 2.3	
Current protocol	294 (26.5)		7.2 ± 1.9	
Operative time(min)		89.3 ± 21.5		< 0.01
≤ 70	253 (22.8)		7.4 ± 4.0	
70–110	646 (58.3)		8.2 ± 4.3	
≥ 110	210 (18.9)		9.5 ± 4.2	
Surgeon				< 0.01
1	297 (26.8)		7.9 ± 4.5	
2	320 (28.9)		8.57 ± 4.1	
3	306 (27.6)		7.4 ± 4.0	
4	186 (16.8)		10.2 ± 3.7	
ROM		91.3 ± 18.0		< 0.01
<90°	344 (35.4)		8.6 ± 4.4	
≥ 90°	583 (64.6)		7.7 ± 4.1	
Complications				< 0.01
Yes	220 (19.8)		11.0 ± 4.2	
No	892 (80.2)		7.6 ± 4.0	
Intraoperative blood loss(ml)		219.8 ± 120.5		< 0.01
≤ 150	291 (41.3)		7.7 ± 4.0	
150–300	287 (40.8)		8.2 ± 4.2	
≥ 300	126 (17.9)		10.6 ± 4.0	
Preoperative Hb(g/L)		127.9 ± 13.7		0.571
≤ 110	108 (9.7)		7.6 ± 4.4	
110–140	786 (70.8)		8.5 ± 4.3	
≥ 140	116 (10.5)		7.3 ± 4.1	
BI Score				0.512
≤ 90	576 (67.6)		7.3 ± 4.0	
>90	276 (32.4)		7.8 ± 4.2	
Day of operation				0.221
Monday	245 (22.0)		8.3 ± 4.4	
Tuesday	236 (21.2)		8.9 ± 4.1	
Wednesday	168 (15.1)		7.6 ± 3.9	
Thursday	212 (19.1)		8.4 ± 4.4	
Friday	210 (18.9)		8.1 ± 4.0	
Type of pay				0.412
Selfpay	577 (60.8)		8.0 ± 4.1	
Medicare	372 (39.2)		8.2 ± 4.4	
Ward				< 0.01
A	923 (83.2)		8.0 ± 4.2	
B	186 (16.8)		9.8 ± 4.1	
Transfusion				< 0.01
Yes	87 (7.8)		11.0 ± 5.2	
No	1025 (92.2)		8.1 ± 4.1	
ASA classification				0.015
I/II	920(82.7)		8.1 ± 3.5	
III/IV	192(17.3)		9.3 ± 4.1	
Comorbidities				0.043
Healthy	674		7.9 ± 4.	
Cardiovascular	85		8.5 ± 3.7	
Neurological	93		9.7 ± 3.2	

*P* < 0.05 was considered statistically significant. *ROM* Range of motion, *BI* Barthel Index. *SD* Standard deviation, *Hb* Hemoglobin

**Fig. 1:**
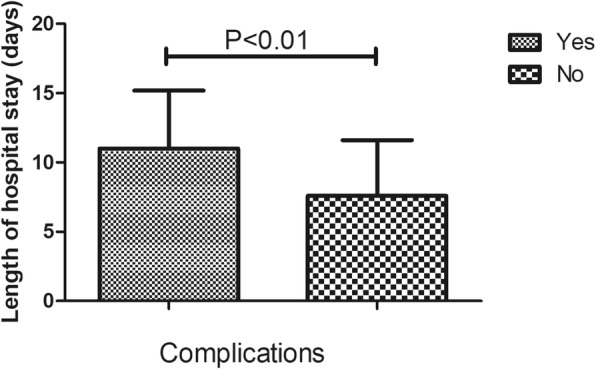
The effect of complications on length of hospital stay

**Fig. 2:**
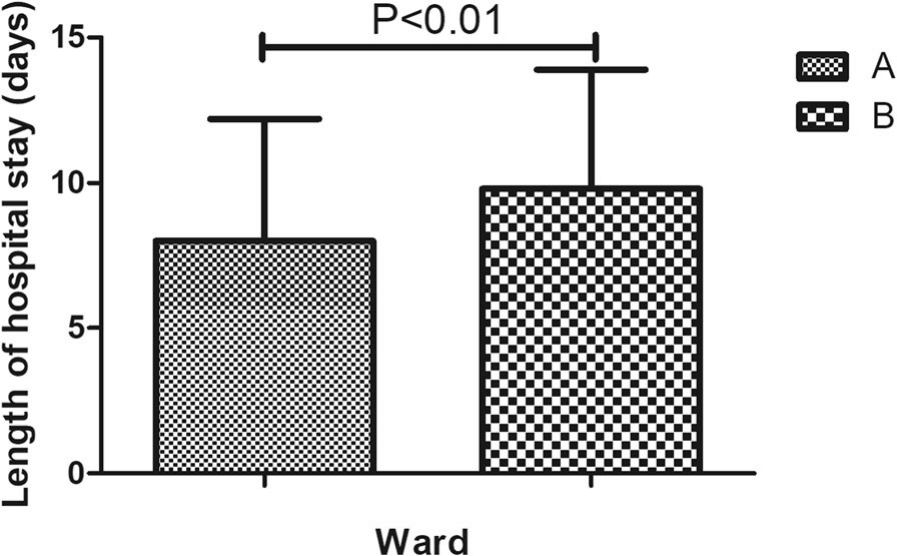
The effect of ward on length of hospital stay

**Fig. 3:**
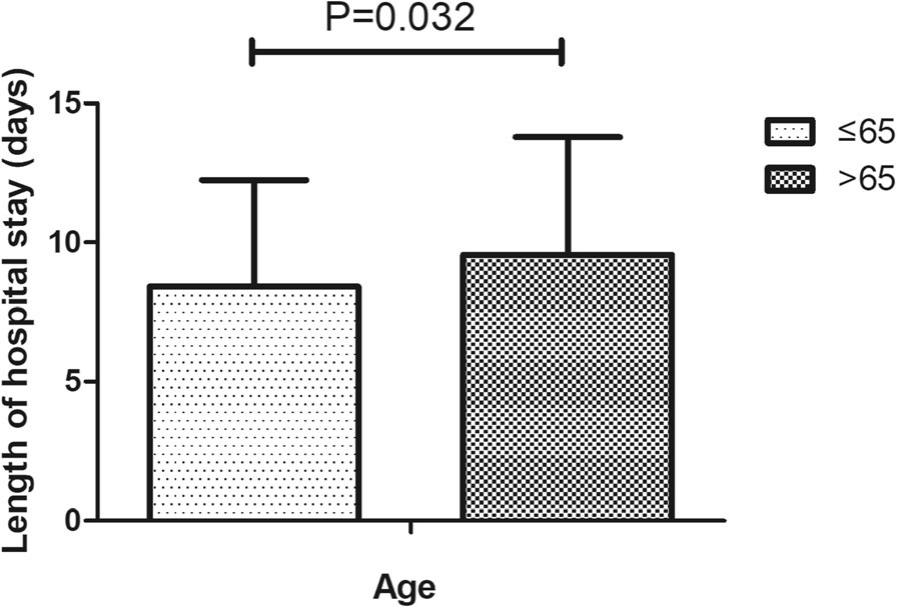
The effect of age on length of hospital stay

**Fig. 4:**
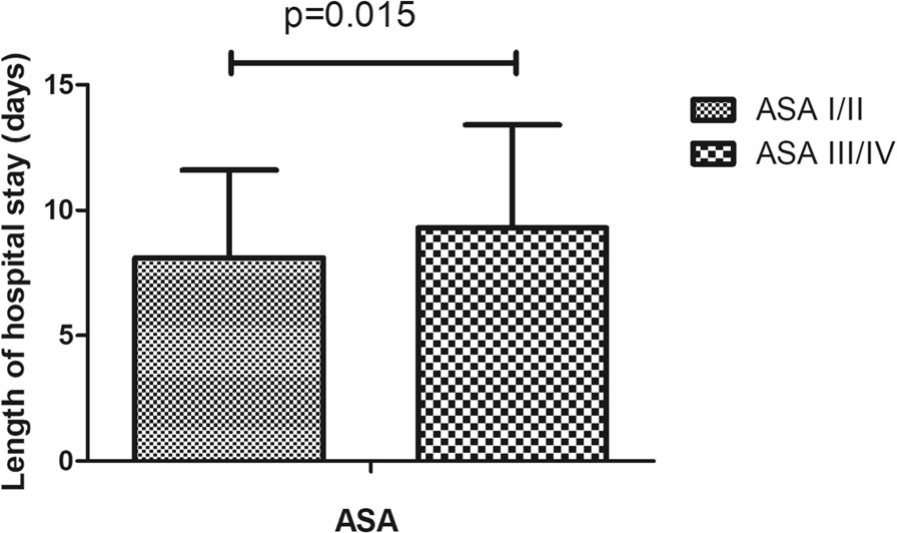
The effect of ASA on length of hospital stay

**Fig. 5:**
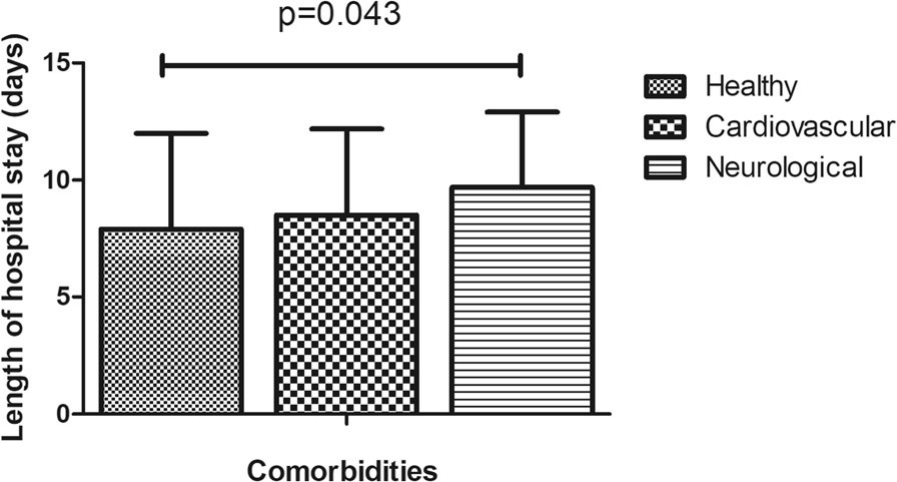
The effect of comorbidities on length of hospital stay

**Fig. 6:**
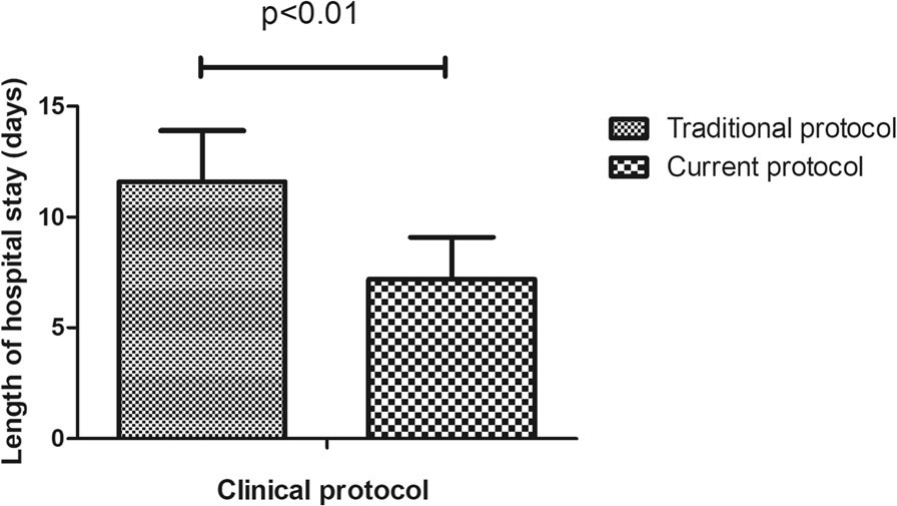
The effect of clinical protocol on length of hospital stay

**Fig. 7:**
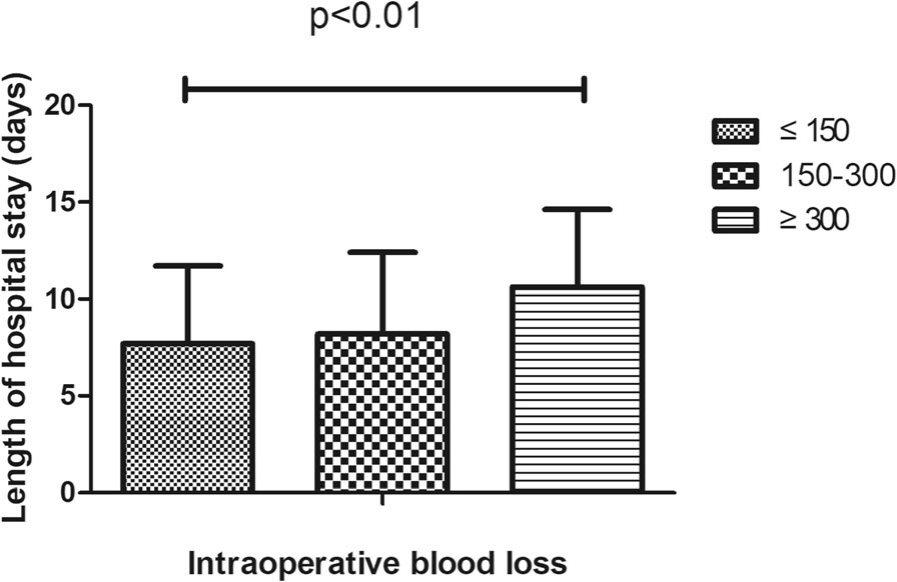
The effect of intraoperative blood loss on length of hospital stay

**Fig. 8:**
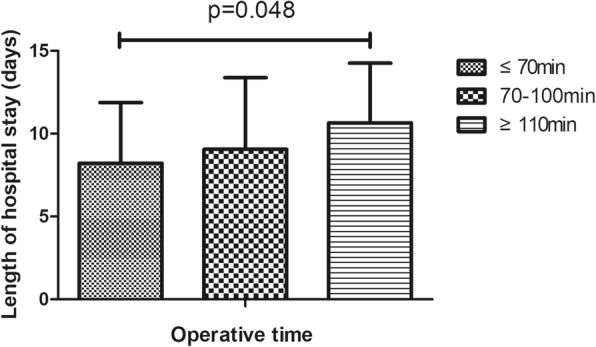
The effect of operative time on length of hospital stay

**Fig. 9:**
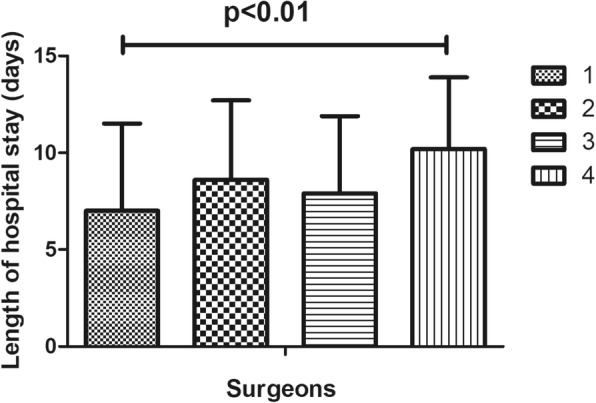
The effect of surgeon on length of hospital stay

In this study, 220 (18.3%) cases of complications were recorded, which mainly included infection, wound exudate, persistent fever and thrombogenesis. Patients with complications stayed in the hospital longer (11.6 days) than patients without complications (7.5 days), as depicted in Fig. 1. Patients in ward B stayed longer (9.8 days) than patients in ward A (8.0 days), as depicted in Fig. 2. Older patients stayed in the hospital longer (> 65 years, 9.5 days) than the younger group (≤65 years, 7.9 days, Fig. 3). Patients with ASA III/IV tended to stay longer (9.3 days) than those with ASA I/II (8.1 days, Fig. 4). Patients with neurological disease stayed in the hospital longer (9.7 days) than healthy patients (7.9 days, Fig. 5), and patients undergoing surgery with the traditional protocol stayed in the hospital longer than those undergoing surgery with the current protocol, as depicted in Fig. 6. A downward trend in the LOS was noted with a decrease in intraoperative blood loss, as depicted in Fig. 7. Similarly, for every increase in operative time by 30 min, the LOS increased by 10.8 to 15.9%, as depicted in Fig. 8. The selection of the surgeon can also influence LOS, as depicted in Fig. 9.

In the univariate analysis, the patient’s age, the ward, the clinical protocol, the operative time, the surgeon, complications, intraoperative blood loss, transfusion, intraoperative blood loss, and ROM showed an association with a prolonged LOS (Table 1). After adjusting for other variables, 9 factors were found to be associated with prolonged length of stay, including complications (*p* < 0.01), the patient’s age (*p* = 0.032), the clinical protocol (*p* < 0.01), intraoperative blood loss (*p* = 0.029), the operative time (*p* = 0.048), the surgeon (*p* = 0.015), the ward (*p* < 0.01), the ASA classification (*p* = 0.045), and comorbidities (*p* = 0.027) (Table 2)**.**

**Table 2:** Binary regression model for perioperative predictors of prolonged length of hospital stay

Variables	*P*-value	Odds Ratio (95%CI)
Age (>65 vs ≤ 65)	0.032	1.98 (0.85 to 1.41)
Clinical protocol (vs current)		
Traditional	< 0.01	6.81 (3.85 to 12.04)
Operative time (≥110 min vs)		
≤ 70 min	0.048	1.96 (0.99 to 3.89)
70-110 min	ns	1.19 (0.86 to 2.69)
Surgeon (vs 4)		
1	0.015	0.39 (0.03 to 0.30)
2	ns	0.77 (0.42 to 1.43)
3	ns	0.86 (0.47 to 1.40)
Comorbidities (vs healty)		
Cardiovascular	ns	1.14 (0.56 to 1.78)
Neurological	0.027	2.13 (2.11 to 3.32)
ASA classification (III/IV vs I/II)	0.045	1.91 (1.01 to 3.59)
ROM (<90°vs ≥ 90°)	ns	1.31 (−0.14 to 0.49)
Transfusion (no vs yes)	ns	0.82 (0.53 to 1,23)
Complications (no vs yes)	< 0.01	0.28 (0.14 to 0.56)
Ward (B vs A)	< 0.01	4.23 (1.76 to 3.14)
Intraoperative blood loss(≥300 ml vs)		
≤ 150	0.029	2.05 (1.08 to 3.92)
150–300	ns	1.71 (0.97 to 3.40)
Preoperative Hb(g/L)	ns	0.98 (0.32 to 1.41)

*P* < 0.05 was considered statistically significant. *ROM* Range of motion, *CI* Confidence interval

## Discussion

In this study, we investigated 16 potential factors and found that 9 factors were associated with a prolonged LOS, including postoperative complications, the patient’s age, the clinical protocol, intraoperative blood loss, the operative time, the surgeon, the ward, the ASA classification, and neurological comorbidities.

Complications were also found to be a contributing factor for prolonged LOS in previous studies [7, 8]. Patients in our centre were not allowed to be discharged from the hospital once complications such as DVT and infection occurred unless the complications were controlled.

Patients in ward A tended to have shorter hospital stays than those in ward B, which is in accordance with the findings in a previous study [13]. In our department, ward A had more trained therapists and surgeons, which partly explains the faster turnover of patients in ward A.

Consistent with the results of other studies, the older group of patients (> 65 years) was likely to stay in the hospital longer than the younger group (≤65 years) [7, 18–20]. Older patients tend to be in worse physical condition and more likely to have postoperative complications [21, 22], which increases the LOS. Furthermore, older patients are more likely to live alone and need caregiver support than are younger patients, and these factors have been shown to be important predictive factors for an extended LOS [6].

The ASA classification is frequently used to define patients’ physical condition [11, 13]. It is reasonable to conclude that patients with ASA III/IV often stay longer than those with ASA I/II, as they require much more time to reach the strict discharge criteria. Additionally, we assessed the influence of comorbidities and found that neurological comorbidities can prolong hospital stays. We speculate that neurological diseases make it difficult for patients to exercise soon after surgery. In contrast to the results of other literature, we did not find a statistically significant association between cardiopulmonary disease and prolonged LOS, which may be due to the small sample sizes.

Beginning in 2014, a new clinical protocol was implemented in our department. Comparing with the traditional clinical protocol, the new one is more reasonable and sound owning to the improvement of some perioperative management protocol, including building a new rehabilitation team and introducing a “cocktail pain control” model. In this study, we investigated whether a good perioperative protocol can change patients’ LOS. Our results showed that people stayed in the hospital for a much shorter period when the protocol was implemented in patients who received TKA surgery. Thus, hospitals in developing countries should develop sound perioperative management plans to shorten LOS and increase the medical resource utilization rate.

We found that a longer operative time is positively correlated with a prolonged LOS, which is in accordance with the results of a study by Ifeoma et al. [11]. An explanation for this result might be that a tourniquet is routinely used for patients undergoing TKAs in our institution. Therefore, when the operative time increased, the tourniquet time increased accordingly. The hypoxia-ischaemia induced by the use of tourniquet may result in the swelling of soft tissue, weakened strength of the quadriceps muscle and pain in the thigh [23, 24], which slows early rehabilitation training and consequently leads to a prolonged LOS; another explanation may be the positive correlation between a prolonged operative time and an increased infection rate after TKAs [25].

We found that increased intraoperative blood loss is associated with a prolonged LOS, which has not been previously reported. A possible reason for this correlation may be that the loss of blood during an operation can make patients feel weak and make it difficult for them to perform early physical exercise.

In this study, we found that surgeons have a positive effect on LOS following TKA, which is consistent with the finding of Monsef et al. [17]. A possible explanation for this result may be that surgeons had different experiences in managing patients during the perioperative period. Additionally, they may have been relaxed or firm when determining whether the patients should be discharged.

In reality, patients’ decisions may affect LOS because they have the right to decide when to leave hospital. However, it is difficult for us to record patients’ preferences and analyse these data. The patients who want stay in the hospital for a longer period may have common traits, such as being discharged to their home, living alone and having no caregiver support, which have been suggested as predictors of a prolonged LOS [26]. Unfortunately, in our database, this information was not recorded. We hope that these patient-related variables will be analysed in the future.

There is no consensus on the contribution of sex to a prolonged LOS. Some researchers have found that females tend to have a longer LOS than males [6, 11, 27]. The authors explained that females are more likely to have higher rates of obesity, postoperative transfusion and postoperative complications. However, some studies did not find statistically significant differences in LOS between the two groups [10, 12]. Similarly, in our study, the difference between males and females was not obvious. However, this result may be caused by the unbalanced sex ratio (1:3). Therefore, more studies should be performed to investigate the effect of sex on LOS. In addition to sex, other variables such as the BI score, the VAS score, ROM, the day of operation, and transfusion were not associated with prolonged LOS in our study.

There are some limitations to this study. First, we split continuous variables into groups based on the sample mean, which is the most common approach used in the literature. Information loss is inevitable, and therefore, the statistical power to detect a correlation may have been weakened. Second, we extracted all data from medical records, and the data for several factors, such as the VAS score, ROM and intraoperative blood loss, may not be accurate; however, bias can be accommodated due to the large sample size. Third, all of the patients analysed were from our institution; thus, the result may not be representative of all hospitals in China.

Despite the above limitations, this study has important clinical value for several reasons. First, this is the first article to explore the association between perioperative variables and LOS following TKAs in China. Moreover, we found nine factors that positively contribute to a prolonged LOS. This result is reliable and meaningful because of the high quality of our centre compared with other orthopaedic clinics in China and the large sample size in this study, even though this is a single-centre study. In addition, all of the data in our study were collected from medical records rather than from a database. Therefore, we could investigate some variables that cannot be retrieved via procedural codes. Finally, 16 variables were included in the present study, which is much more than the number of variables included in other similar studies. Importantly, the sample size was sufficiently large to generate a reliable statistical outcome. Although fast-track protocols are prevalent in developed countries, the long LOS model is common among developing countries [7, 8]. Therefore, it is essential to explore factors that can potentially reduce the LOS following TKAs in developing countries.

## Conclusion

We found that complications, the patient’s age, the clinical protocol, intraoperative blood loss, the operative time, the surgeon, the ward, the ASA classification, and neurological comorbidities are nine predictive factors contributing to a prolonged LOS. These factors can be used to provide counselling to patients and to further optimize perioperative protocols for the patients at risk for an increased LOS following TKAs.

## Data Availability

The datasets used and/or analyzed during the current study will be available from author XXS on a reasonable request.

## Abbreviations

ASA: American Society of Anesthesiologists
DVT: Deep vein thrombosis
LOS: Length of hospital stay
OA: Osteoarthritis
RA: Rheumatoid arthritis
ROM: Range of motion
TKA: Total knee arthroplasty
